# Renoprotective Impacts of *Inonotus obliquus* Ethanol-Ethyl Acetate Extract on Combined Streptozotocin and Unilateral Nephrectomy-Induced Diabetic Nephropathy in Mice

**DOI:** 10.3390/ijms24054443

**Published:** 2023-02-23

**Authors:** Kuang-Hsing Chiang, Yi-Chun Chiu, Noi Yar, Yu-Chun Chen, Chia-Hui Cheng, Yi-Chien Liu, Chia-Yu Chang, Jiunn-Jye Chuu

**Affiliations:** 1 Taipei Heart Institute, Taipei Medical University, Taipei 11031, Taiwan; 2 Division of Cardiology and Cardiovascular Research Center, Taipei Medical University Hospital, Taipei 11031, Taiwan; 3 Department of Internal Medicine, School of Medicine, College of Medicine, Taipei Medical University, Taipei 11031, Taiwan; 4 Graduate Institute of Biomedical Electronics and Bioinformatics, National Taiwan University, Taipei 10617, Taiwan; 5 Division of Urology, Department of Surgery, Taipei City Hospital, Taipei 10341, Taiwan; 6 Urological Research Center, National Yang Ming Chiao Tung University, Taipei 10662, Taiwan; 7 Department of Exercise and Health Sciences, University of Taipei, Taipei 11153, Taiwan; 8 Department of Biotechnology and Food Technology, College of Engineering, Southern Taiwan University of Science and Technology, Tainan 71005, Taiwan; 9 Department of Neurology, Chi-Mei Medical Center, Tainan 71004, Taiwan; 10 Center for General Education, Southern Taiwan University of Science and Technology, Tainan 71005, Taiwan; 11 Pharmacy Department, Wei-Gong Memorial Hospital, Miaoli 35159, Taiwan

**Keywords:** diabetes nephropathy, *Inonotus obliquus*, streptozotocin, nephrectomy, TGF-β, α-SMA

## Abstract

Diabetes nephropathy (DN) is one of the most common causes of end stage renal disease (ESRD) globally. Medication options to stop or slow the progression of chronic renal disease (CKD) are limited, and patients with DN remain at a high risk of developing renal failure. *Inonotus obliquus* extracts (IOEs) of Chaga mushroom have been shown to have anti-glycemic, anti-hyperlipidemia, antioxidant, and anti-inflammatory effects against diabetes. In this study, we examined the potential renal protective role of an ethyl acetate layer after water-ethyl acetate separation from *Inonotus obliquus* ethanol crude extract (EtCE-EA) from Chaga mushrooms in diabetic nephropathy mice after preparation with 1/3 NT + STZ. Our data showed that treatment with EtCE-EA can effectively regulate blood glucose, albumin-creatinine ratio, serum creatinine, and blood urea nitrogen (BUN) levels, and it can improve the renal damage in 1/3 NT + STZ-induced CRF mice with an increase in concentration (100, 300, and 500 mg/kg). In the immunohistochemical staining test, EtCE-EA can effectively reduce the expression of TGF-β and α-SMA after induction according to the increase in the concentration (100 mg/kg, 300 mg/kg), thereby slowing down the degree of kidney damage. Our findings demonstrate that EtCE-EA could provide renal protection in diabetes nephropathy, possibly due to the decreased expression of transforming growth factor-β1 and α-smooth muscle actin.

## 1. Introduction

Chronic kidney disease (CKD) is a global public health problem, and its prevalence and incidence have significantly increased in the past two decades [1]. The global burden of CKD is rapidly increasing, and it is expected to become the fifth most common cause of years of life lost globally by 2040 [2]. The prevalence of chronic kidney disease (CKD) has increased in recent decades alongside an increase in diabetes and hypertension, the main drivers of CKD [3]. Despite showing a decline in mortality due to the advancements in medical treatment in patients with end-stage kidney disease (ESKD), it has still remained one of the leading causes of death worldwide [4]. Globally, approximately 850 million people were reported to be affected by CKD in 2017 [3]. In 2010, 2.6 million people worldwide received renal replacement therapy, yet an estimated equivalent number died in the same year owing to a lack of access to dialysis and transplantation, particularly in low-income countries [5]. This contrasts with that of other major chronic illnesses, such as cardiovascular and respiratory disorders, whose effects on mortality are decreasing.

CKD can be defined as a persistent presence of kidney damage or decreased kidney function for more than three months, irrespective of the cause, and classified by cause, GFR category (G1–G5), and albuminuria category (A1–A3) [6]. Globally, diabetes and/or hypertension are the most prevalent causes of CKD [7]. The rising prevalence of type 2 diabetes is causing an increase in the number of patients with ESKD caused by diabetic nephropathy (DM) [8]. The presence of CKD is markedly higher in patients with diabetes. Diabetic nephropathy (DN) is the most common complication of diabetes mellitus, affecting approximately 40% of patients with type II diabetes, and is a leading cause of end-stage renal disease (ESRD) worldwide in the last decade [9]. Therefore, the management of diabetes is a major component of CKD prevention. In type 2 DM, hyperglycemia leads to the elevation of key pathogeneses of renal damage, such as oxidative stress, insulin resistance, and pro-inflammatory cytokines, and glycemic control may delay the development and progression of CKD [10].

Moreover, DN is not the only cause of CKD in diabetes patients. The prevalence of nondiabetic kidney disease (NDKD) caused by factors irrelevant to DM, such as immunoglobulin A nephropathy (IgA N) and membranous nephropathy (MN), varies from 12 to 79% in adults with DM [11]. In contrast to diabetic nephropathy, many kinds of non-diabetic kidney disease can be effectively treated (e.g., glomerulonephritis with immunosuppressive medication) [12]. New anti-diabetes agents (glucagon-like peptide-1 receptor (GLP-1R), agonists, dipeptidyl peptidase-4 (DPP-4) inhibitors, and sodium-glucose transporter-2 (SGLT-2) inhibitors) were found to have renal protective effects via anti-hypertensive, hemodynamic stabilization, anti-inflammatory, and anti-oxidative actions [13,14,15,16]. Modifiable risk factors for the development and progression of CKD in diabetes patients include systemic hypertension, proteinuria, and metabolic factors, such as insulin resistance, dyslipidemia, and hyperuricemia, etc. [17]. Thus, regardless of etiology, either DN or NDKD, control of glucose, hypertension, diet, and body weight is essential in the prevention of kidney disease in diabetes patients. In addition to glucose-lowering therapies, lifestyle interventions, including diet are associated with clinically significant improvements in diabetes control [18]. Studies have suggested that functional foods may improve hyperglycemia by modulating carbohydrate and lipid metabolism in adipose tissues and also by reducing oxidative stress and inflammatory processes, and subsequently, they could prevent the development of diabetes nephropathy [19].

Nowadays no definitive drug is available to stop or slow down the progression of chronic renal disease, and the medication options are influenced by the presence of comorbid diseases the patients have and their individual risk of complications. The *Inonotus obliquus* mushroom, also known as Chaga, mainly grows in cold areas (for example in northeast China, northern Europe, and Russia) and is used traditionally in the treatment of diabetes, cardiovascular disease, and gastrointestinal diseases [20]. To date, a few studies have suggested the significant therapeutic potential of *Inonotus obliquus* extracts (IOEs), which have been shown to have therapeutic effects against diabetes via multiple pathways including anti-glycemic, anti-hyperlipidemia, antioxidant, and anti-inflammatory effects in various studies [21]. The low molecular weight of IOEs has been shown to restore the integrity of the glomerular capsules, increase the number of glomerular mesangial cells, and protect renal tubular cells against STZ + AGEs-induced glucotoxicity in diabetic mice [22]. However, there has been no work so far presenting scientific findings on the renal protective effect of IOE in CKD patients. Our study aims to explore the potential possibility of using IOE as renal protective medication in CKD patients.

## 2. Results

**Figure 1:**
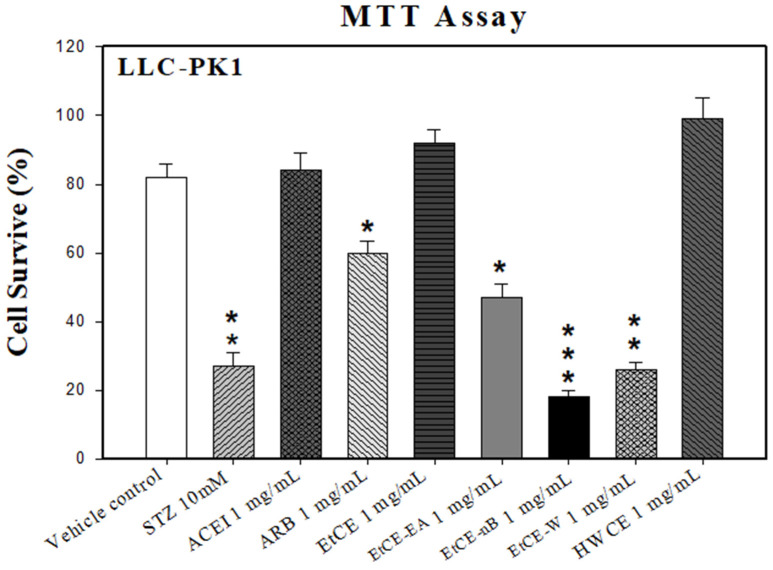
Viability of *Inonotus obliquus* fruit bodies extracts (IOEs) in LLC-PKI cells. The LLC-PK1 cells were cultured in Nine groups; vehicle control, STZ (10 mM), ACEI (1 mg/mL), ARB (1 mg/mL), EtCE: Ethanol crude extract of *Inonotus obliquus* fruit bodies (1 mg/mL), EtCE-EA: Ethyl acetate layer after water-ethyl acetate separation from ethanol crude extract (1 mg/mL), EtCE-nB: n-butanol layer after water-n-butanol separation from ethanol crude extract (1 mg/mL), EtCE-W: water layer after water-n-butanol separation from ethanol crude extract (1 mg/mL) and HWCE: hot water crude extract of *Inonotus obliquus* fruit bodies (1 mg/mL), for 72 h alone and cell viability was observed. The cell cytotoxicity was determined by MTT. Each value represents the mean ± SD of three replicated experiments, and the results are expressed as population growth. *: *p* < 0.05, **: *p* < 0.01 and ***: *p* < 0.001 as compared with the vehicle control.

Before evaluating the effect of *Inonotus obliquus* extracts (IOEs) on STZ-induced porcine proximal tubular (LLC-PK1) cells, a cell viability assay was performed to determine the appropriate concentration of IOEs for further STZ-induced improvement assays. The results showed better viability in the ACEI (1 mg/mL), EtCE (1 mg/mL), and HWCE (1 mg/mL) compared to the vehicle control (*p* > 0.05, *p* > 0.05 and *p* > 0.05, respectively) while more cytotoxicity and decreased viability were observed in EtCE-EA (1 mg/mL), EtCE-nB (1 mg/mL), and EtCE-W (1 mg/mL) compared to the vehicle control (*p* < 0.05, *p* < 0.001, and *p* < 0.01, respectively) at 72 h (Figure 1).

**Figure 2:**
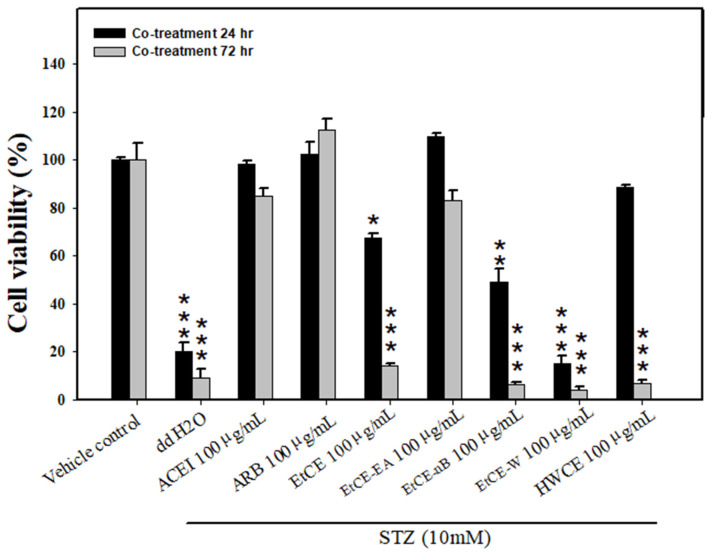
Efficacy of *Inonotus obliquus* fruit bodies extracts (IOEs) protection from STZ-induced cell toxicity in LLC-PK1 cells. Vehicle control (ddH2O alone), ddH2O, ACEI (100 μg/mL), ARB (100 μg/mL), EtCE: Ethanol crude extract of *Inonotus obliquus* fruit bodies (100 μg/mL), EtCE-EA: Ethyl acetate layer after water-ethyl acetate separation from ethanol crude extract (100 μg/mL), EtCE-nB: n-butanol layer after water-n-butanol separation from ethanol crude extract (100 μg/mL), EtCE-W: water layer after water-n-butanol separation from ethanol crude extract (100 μg/mL) and HWCE: hot water crude extract of *Inonotus obliquus* fruit bodies (100 μg/mL) after 24 h STZ (10 mM) treatment and the protective effects from cytotoxicity were determined by MTT in LLC-PK1 cells at 24 and 72 h. Each value represents the mean ± SD of three replicated experiments, and the results are expressed as population growth. *: *p* < 0.05, **: *p* < 0.01 and ***: *p* < 0.001 as compared with the vehicle control.

In order to further simulate the safe dose of induced animals, renal tubular epithelial cells (LLC-PK1) in vitro were treated with STZ (10 mM) at 24 h and 72 h to cause cellular injury. The proximal renal tubular cells of pigs were treated with IOEs for 24 h after STZ 10 mM injury. At either 24 h or 72 h, only the EtCE-EA (100 μg/mL) group, similar to the ACEI (100 μg/mL) and ARB (100 μg/mL), had a better survival rate when compared to the vehicle control (*p* > 0.05, *p* > 0.05 and *p* > 0.05, respectively). However, the remaining IOEs, including the EtCE (100 μg/mL), EtCE-nB (100 μg/mL), EtCE-W (100 μg/mL), and HWCE (100 μg/mL) groups, revealed low cell viability after being co-treated with STZ (10 mM) (*p* < 0.001, *p* < 0.001, and *p* < 0.001, respectively) at 72 h. Each value represents the mean ± SE of three replicated experiments, and the results are expressed as population growth (control as 100%) (Figure 2).

**Figure 3:**
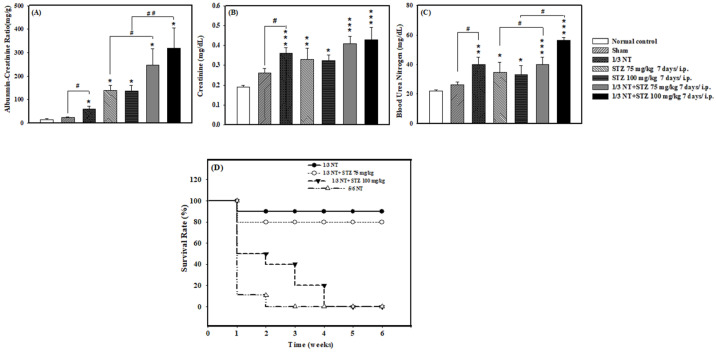
Renal function analysis of mouse models of chronic renal failure. (**A**) Urine albumin-creatinine ratio (ACR), (**B**) Creatinine blood test, (**C**) Blood Urea Nitrogen (BUN), and (**D**) Survival rates were detected in the animal model of chronic renal failure. Normal: native control; Sham: vehicle control; 1/3 NT: residual 2/3 kidney animal model; 5/6 NT: residual 1/6 kidney animal model. The data are expressed as mean ± SE of five mice. *: *p* < 0.05, **: *p* < 0.01 and ***: *p* < 0.001 as compared with the normal control at two weeks. ^#^: *p* < 0.05, ^##^: *p* < 0.01 as compared with STZ alone at the two-week trial.

In order to achieve the ideal renal injury index value in this animal model, the combined induction of the chemical drug STZ at a medium dose (75 mg/kg) and high dose (100 mg/kg) was done at one week after the operation. Urinary albumin to creatinine ratio (ACR) has been used as the preferred indicator for quantifying albuminuria in terms of biochemical values and included in the indicators for assessing the risk of renal failure. The experimental results of 1/3 NT + STZ 75 mg/kg compared with STZ 75 mg/kg reached the expected index Albumin-Creatinine Ratio of 200 mg/g or more while the result of 1/3 NT + STZ 100 mg/kg compared with STZ 100 mg/kg has increased ACR to more than 300 mg/g (*p* < 0.05 and *p* < 0.01, respectively) (Figure 3A) with severe proteinuria, which destroyed glomerular and renal tubular cells in the kidney, making it from chronic renal failure to early renal failure. From blood Creatinine and Blood Urea Nitrogen values, the 1/3 NT + STZ 100 mg/kg group showed the most severe damage, followed by 1/3 NT + STZ 75 mg/kg (*p* < 0.001 and *p* < 0.001, respectively) (Figure 3B,C). In Figure 3D, the survival rate of the 1/3 NT + STZ 100 mg/kg group was about 20% in the third week, and that of the 5/6 NT operation group in the first week was about 10%. Chronic renal failure models all lead to weight loss. Thus, the 1/3 NT + STZ 75 mg/kg group with a survival rate of 80% was selected as the animal model for the follow-up experiment.

**Figure 4:**
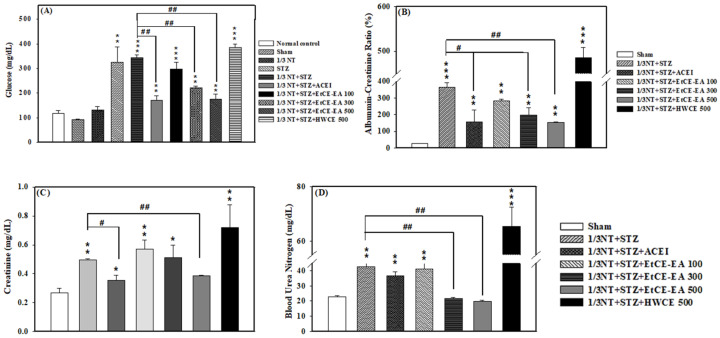
Blood samples were collected from the retro-orbital sinus of CRF mice in each group. (**A**) The fasting blood glucose test, (**B**) Urine albumin-creatinine ratio (ACR), (**C**) Creatinine blood test, and (**D**) Blood urea nitrogen (BUN) levels were measured at two weeks including 1/3 NT +STZ alone, 1/3 NT + STZ following ACEI (20 mg/kg), EtCE-EA: Ethyl acetate layer after water-ethyl acetate separation from ethanol crude extract (100, 300 and 500 mg/kg) and HWCE: hot water crude extract of *Inonotus obliquus* fruit bodies (500 mg/kg) treatments as compared to the sham group. Sham: vehicle control; ACEI (Selective ACE Inhibitor); 1/3 NT: residual 2/3 kidney animal model. The data are expressed as mean ± SD of five mice. *: *p* < 0.05, **: *p* < 0.01 and ***: *p* < 0.001 as compared with the sham control at two weeks. ^#^: *p* < 0.05, ^##^: *p* < 0.01 as compared with 1/3 NT + STZ alone at the two-week trial.

Two weeks after dosing, we first measured fasting blood glucose (Figure 4A), mainly to observe the changes in chronic kidney disease. After induction of renal failure in experimental mice, due to the physical damage of the surgical side of the kidney, the other side will have compensatory hypertrophy, glomerular sclerosis, and functional decline. After administration of IOEs, we tested whether it can improve the oxidative damage of STZ to pancreatic β cells and cause hyperglycemia in vivo. We found that the treatment group with the IOE, EtCE-EA (300 and 500 mg/kg) can effectively utilize the glucose in the body. On the contrary, HWCE (500 mg/kg) group failed to effectively regulate blood sugar compared to the 1/3 NT + STZ alone (*p* < 0.01, *p* < 0.01, and *p* > 0.05, respectively) (Figure 4A). The ratio of albumin to creatinine in urine was observed with Albumin-Creatinine Ratio (Figure 4B) in urine biochemical values. We also found that EtCE-EA (300 and 500 mg/kg) can effectively improve the discharge of proteinuria caused by renal damage compared to the 1/3 NT + STZ alone (*p* < 0.05 and *p* < 0.01, respectively); however, the HWCE (500 mg/kg) group, still failed to effectively improve the renal damage caused by chronic renal failure and the damage degree is more serious than ACEI group (*p* > 0.05 and *p* < 0.05, respectively) (Figure 4B). At the same time, the blood creatinine (Figure 4C) and blood urea nitrogen (Figure 4D) were observed, and the results showed the EtCE-EA, according to the increase of its concentration (100 mg/kg, 300 mg/kg, 500 mg/kg), can effectively improve the abnormal metabolism caused by chronic renal failure in blood creatinine (*p* > 0.05, *p* > 0.05, and *p* < 0.01, respectively) and blood urea nitrogen (*p* > 0.05, *p* < 0.01, and *p* < 0.01, respectively) compared to the 1/3 NT + STZ alone. On the other hand, the HWCE (500 mg/kg) group is not effective in improving the damage to the kidney after 1/3 NT + STZ induction in blood creatinine (*p* > 0.05) and blood urea nitrogen (*p* > 0.05) (Figure 4C,D).

**Figure 5:**
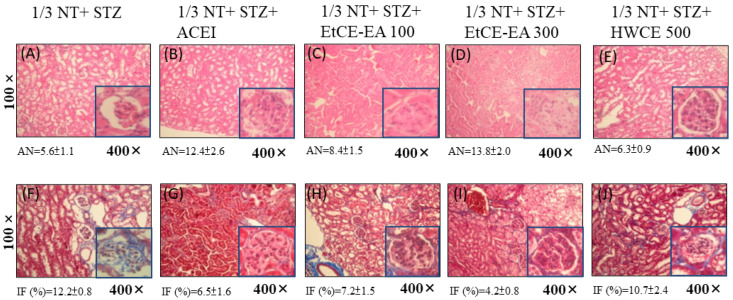
Histopathological study after *Inonotus obliquus* fruit bodies extracts (IOEs) treatment on the renal cortex in CRF mice. Treatment with ACEI (20 mg/kg), EtCE-EA: Ethyl acetate layer after water-ethyl acetate separation from ethanol crude extract (100 and 300 mg/kg) and HWCE: hot water crude extract of *Inonotus obliquus* fruit bodies (500 mg/kg) for two consecutive weeks was given in CRF mice with 1/3 NT + STZ-induced diabetic nephropathy, and histopathology of the renal cortex was studied using H&E stain (**A**–**E**) and Masson’s trichrome stain (**F**–**J**), respectively in sections of the CRF kidney under magnification 100× and 400×. The podocytes of glomerulus in different groups under a light microscope (×400). The average number (AN) of foamy podocytes/glomerulus were calculated. The interstitial fibrosis, IF (% positive) were estimated. Data are presented as means  ±  SEM (n  =  5). For each animal (n = 5 for each group), all glomeruli (about 25–30) on another part of a kidney after unilateral nephrectomy sections were counted.

In the 1/3 NT + STZ plus EtCE-EA (300 mg/kg) (Figure 5D), the focal glomerulus remained relatively intact and numerous with hematoxylin and eosin staining. In the 1/3 NT + STZ plus ACEI (20 mg/kg) (Figure 5G), the glomerulus remained relatively intact with positive collagen staining; weak collagen staining was present in the tubules and the interstitium. In 1/3 NT + STZ treated mice (Figure 5F), obvious mesangial matrix accumulation with diffuse collagen fibril deposition in different compartments was observed. In 1/3 NT + STZ plus EtCE-EA (300 mg/kg) treated mice (Figure 5I), the matrix accumulation and collagen staining were less severe than those in the control group (1/3 NT + STZ alone). In 1/3 NT + STZ + HWCE (500 mg/kg) treated mice (Figure 5J), obvious mesangial matrix accumulation and diffuse collagen staining within renal tissues were observed. It can be seen from the histopathological section of the renal corpus with H&E stain that after induction, the accumulation of renal interstitium was less obvious in the ACEI (20 mg/kg) group, and the morphology of the glomerulus was similar to that of the induction group (1/3 NT+ STZ) (Figure 5A) and almost complete (Figure 5B). The IOE, EtCE-EA group, according to the increase of its concentration (100 mg/kg and 300 mg/kg), can effectively improve the induced glomerular atrophy and interstitial accumulation (Figure 5C,D). We examined sections of CRF mouse renal cortex using Masson’s trichrome (MT) staining to detect the severity of overt nephropathy indicated by collagen fibril deposition in glomeruli, tubules, and interstitium (5F–J). As shown in Figure 5F, in the slices of the induction group without treatment (1/3NT + STZ alone), there was a large amount of collagen deposition in the atrophic interstitium of the glomerulus. While in EtCE-EA group (100 mg/kg, 300 mg/kg), the deposition of collagen showed a tendency to slow down due to the increase in concentration (Figure 5H,I). However, in the HWCE (500 mg/kg) group (Figure 5J), although there was no slowing and improvement, it was worse when compared with the pathological state of the ACEI group (Figure 5G).

**Figure 6:**
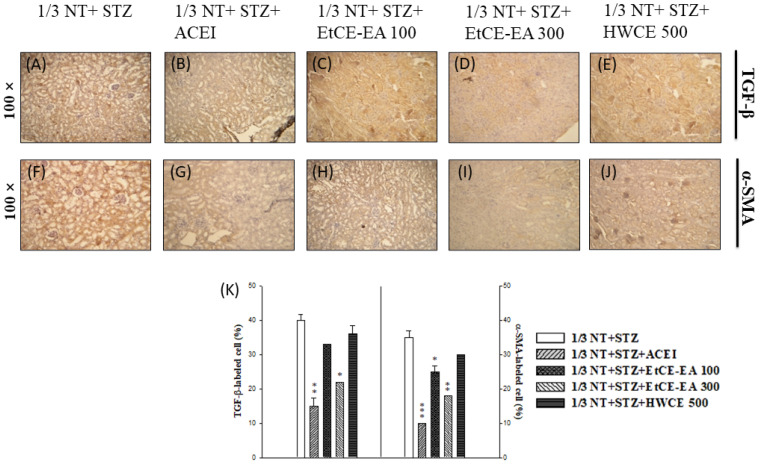
Immunohistochemical staining (TGF-β and α-SMA) after *Inonotus obliquus* fruit bodies extracts (IOEs) treatment in CRF kidney. Treatment with ACEI (20 mg/kg), EtCE-EA: Ethyl acetate layer after water-ethyl acetate separation from ethanol crude extract (100 and 300 mg/kg) and HWCE: hot water crude extract of *Inonotus obliquus* fruit bodies (500 mg/kg) for two consecutive weeks was given in CRF mice with 1/3 NT + STZ-induced diabetic nephropathy, and histopathology of the renal cortex was studied using immunohistochemical staining of TGF-β (**A**–**E**) and α-SMA (**F**–**J**) expression, respectively at two weeks. The representative staining photomicrographs of TGF-β-positive cells and α-SMA -positive cells in each section were examined under light microscopy (magnification ×400). After 5 random fields from each section were captured, the number of TGF-β-positive cells and α-SMA -positive cells (in brown) in the corresponding proliferative zone (glomerulus area) within each field was computed by using Image Pro Plus software (Version 5.1) and was compared to the 1/3 NT + STZ (**K**). *: *p* < 0.05, **: *p* < 0.01 and ***: *p* < 0.001 as compared with the normal control at two weeks.

In the immunohistochemical staining test, the expression levels of renal fibrosis factors TGF-β (Figure 6A–E) and α-SMA (Figure 6F–J) were analyzed. According to the increase in the concentration (100 mg/kg, 300 mg/kg), EtCE-EA can effectively reduce the expression of TGF-β and α-SMA after induction, thereby slowing down the degree of kidney damage. While in the EtCE-EA (300 mg/kg) group, the expression amount is close to the expression amount of the ACEI (20 mg/kg) group, which shows that it can effectively inhibit the expression amount and slow down the level of fibrosis. Finally, by quantifying the positive cells (%) marked by TGF-β and α-SMA, it can be determined that the EtCE-EA (300 mg/kg), rather than EtCE-EA (100 mg/kg) and HWCE (500 mg/kg), significantly inhibits the formation of α-SMA myofibroblasts (*p* < 0.05, *p* > 0.05, and *p* > 0.05, respectively) by reducing the expression of TGF-β (*p* < 0.01, *p* < 0.05, and *p* > 0.05, respectively); therefore, it can improve the phenomenon of renal deterioration in the animal model of chronic renal failure compared with the ACEI group (*p* < 0.01, *p* < 0.001, respectively) (Figure 6K), thus confirming the therapeutic potential of *Inonotus obliquus* fruit bodies extract (IOE), the EtCE-EA in chronic renal failure.

## 3. Discussion

In recent years, growing interest was seen in the use of *Inonotus obliquus* extracts (IOEs) for the treatment of diabetes and renal disease. Still, a limited number of studies have demonstrated the therapeutic effectiveness of IOEs in the treatment of diabetic nephropathy. In this study, treatment with the extraction of EtCE-EA (Ethyl acetate layer after water-ethyl acetate separation from *Inonotus obliquus* ethanol crude extract) can effectively improve the renal damage in 1/3 NT + STZ-induced CRF mice with an increase in concentration (100, 300, and 500 mg/kg). An effective reduction in the expression of TGF-β and α-SMA after induction and subsequent slowing of the degree of kidney damage was observed.

Chaga fungus was proven to possess antioxidant, hypoglycemic, hypolipidemic, and anti-tumor properties, and the use of Chaga extracts, IOEs in the treatment of diabetes and kidney disease has been examined by several scientific studies. Chaga extracts contain several compounds such as polysaccharides, triterpenes, and polyphenols [23]. The exact mechanisms of action for the hypoglycemic effect of IOEs have not been reached conclusion. So far, it has been described that I. obliquus polysaccharides in streptozotocin (STZ)-induced diabetic rats reduced blood glucose levels and restored the structure of β-cells after diabetes-induced cellular damage [24]. Wang et al. reported that *Inonotus obliquus* polysaccharides enhanced the serum levels of insulin and alleviated the metabolic derangement of glucose enzymes in the STZ-induced diabetic mice model [21]. Another study has found that the ingestion of *Inonotus obliquus* polysaccharide had improved serum insulin levels, moderately expanded the pancreatic islets, and reduced pancreatic injuries in alloxan-induced diabetic mice [25]. One of the main ingredients of *Inonotus obliquus* extract, Trametenolic acid (TA), was also recently reported to have a renal protective effect in diabetic nephropathy by relieving oxidative stress and inflammation via Nrf2/HO-1 and NF-κB signaling pathways [26]. However, the role of *Inonotus obliquus* ethyl acetate extract in the protection of renal impairment caused by diabetes still remains uncertain. We proposed that the critical issue could be the preparation of IOE, and our method with ethyl acetate extract was evaluated. Further, transforming growth factor-β (TGF-β) is a potent stimulator that drives fibrosis, and the downregulation of TGF-β has been found to significantly limit the fibrotic process in chronic kidney disease [27,28,29]. The induction of alpha-smooth muscle actin (α-SMA), a smooth muscle cell marker protein, increases extracellular matrix deposition and glomerulosclerosis, and a high α-SMA expression in kidneys is a hallmark of tubular epithelial-myofibroblast trans-differentiation [30]. Our results demonstrated dose-related improvements in blood glucose and renal function results with the EtCE-EA. We also found that EtCE-EA had a renal protective function through the downregulation of both TGF-β1 and α-SMA. These findings suggest that EtCE-EA could provide renal protection in diabetic mice with severe CKD, which requires additional clinical validation.

Animal models are crucial for pathological and clinical research on disease treatment therapies to understand therapeutic outcomes and drug safety. The process of selection of the animal model is a very intricate part as many factors need to be considered to reproduce the disease and pathology at the same level as that of humans [31]. Streptozotocin is one of the most commonly used substances to induce diabetes in experimental mice [32]. Also known as subtotal nephrectomy, 5/6 nephrectomy has been a widely used model for studying CKD [33]. However, this model causes a great risk of hemorrhage and infection during surgery and high animal mortality [34]. In the present study, the selected animal model was created with the combination of STZ and 1/3 nephrectomy to closely mimic chronic and more severe renal injury and to signify the protective effect of EtCE-EA.

Fasting blood glucose, the albumin-creatinine ratio (ACR), serum creatinine levels, and serum blood urine nitrogen (BUN) levels are the most used biochemical parameters to estimate the progression of renal disease and diabetes control. In our study, treatment with EtCE-EA can effectively regulate blood sugar, ACR, serum creatinine, and BUN according to the increase of its concentration (100 mg/kg, 300 mg/kg, 500 mg/kg) while HWCE treatment has caused more severe damage. According to previous studies, different extraction methods have exhibited different drug components and properties [35]. It is preliminarily inferred that the hot water extraction of Chaga mushroom directly dissolved potential substances that accelerate the deterioration and failure of the kidneys. Cases of oxalate-induced nephropathy from long-term ingestion of Chaga mushroom powder were reported in recent studies [36]. Oxalate, an organic acid found in Chaga mushroom extracts, can cause nephropathy from excessive intake [37]. It is found in high concentrations especially in water extracts of Chaga mushroom than in ethanolic extracts [38]. Therefore, noting the oxalate concentration of Chaga mushroom extracts and methods of extraction may be important in order to avoid or lessen the risk of oxalate nephropathy. Xu and co. reported that an ethanol extract of the dry matter of a culture broth of I. obliquus has shown significant anti-hyperglycaemic, as well as anti-lipid peroxidative effects, against alloxan-induced diabetic mice [34].

In this study, we examined the potential renal protective role of EtCE-EA from Chaga mushroom in mice after preparation with 1/3 NT + STZ [36]. To demonstrate the renal protective role of EtCE-EA beyond its anti-diabetic effect, an animal model was systematically established to mimic pathophysiology of severe renal impairment in diabetes patients. In natural medicine, unlike conventional medicine, methods of preparation have an impact on the function/chemical property of the extracts. Thus, we further investigated the efficacy of IOEs by different methods. We believe that this study has provided strong evidence that IOE plays a renal protective role in diabetes nephropathy, which may be at least partially attributed to the decreased expression of transforming growth factor-β1 and α-smooth muscle actin, deepening the understanding functions of IOE in diabetic nephropathy.

## 4. Materials and Methods

### 4.1. Chemicals and Reagents

Culture medium RPMI-1640, fetal bovine serum, sodium bicarbonate, l-glutamine, and 0.05% trypsin-EDTA were from Gibco Ltd. Streptozotocin (STZ) was from Sigma (Saint Louis, MO, USA). *Inonotus obliquus* fruit body was produced by TCM Biotech International Corp. (Xizhi District, New Taipei, Taiwan). Selective ACE Inhibitor was produced by Taiwan Tanabe Seiyaku Co., Ltd. (Nangang, Taipei, Taiwan). Renal tubular cells, LLC-PK1 were purchased from Food Industry Research and Development Institute (Eastern Hsin Chu, Taiwan). Culture medium RPMI-1640 was produced by Thermo Fisher Scientific Inc. (Waltham, MA, USA). Thiazolyl Blue Tetrazolium Bromide was produced by Sigma–Aldrich Inc. (Burlington, MA, USA). The rabbit polyclonal antibodies-TGF-β, Rabbit α-SMA Polyclonal Antibody were from Santa Cruz Biotechnology, Inc. (Delaware Ave, Santa Cruz, CA, USA).

### 4.2. Preparation of Inonotus obliquus Body Extract

An amount of 10 g of *Inonotus obliquus* fruiting body was taken out again; 100 mL of 95% ethanol was added to extract for 24 h, and the suspension was obtained by centrifugation. The suspension was placed in an oven at 60 °C for 6 h, and concentrated to 10 mL to obtain the EtCE, ethanol crude extract of *Inonotus obliquus* fruiting bodies. Then, the EtCE was partitioned between water and ethyl acetate solution (*v*/*v* = 1:1 ratio) and centrifuged to obtain the ethyl acetate layer (EtCE-EA) and water layer. The water layer was partitioned between water and n-butanol (*v*/*v* = 1:1 ratio) to finally gain the n-butanol layer (EtCE-nB) and the water layer (EtCE-W), respectively. Then, the water layer was mixed with n-butanol (*v*/*v* = 1:1 ratio) to finally obtain the n-butanol layer and the water layer. Additionally, the hot water crude extract of *Inonotus obliquus* fruit bodies (HWCE) was also prepared (*w*/*v* = 1:10 ratio). These fractions above were placed in an oven at 60 °C for 6 h, and after concentrating the suspension to 10 mL, freeze-drying was performed to obtain the extracts of *Inonotus obliquus* fruiting body (IOEs). The MWs of HWCE are closely correlated with their functional bioactivities, and HWCE active polysaccharide mostly ranged 780 kDa (Mw), identified with gel permeation chromatography analysis. Meanwhile, the HWCE extract contained 17.11 mg/mL of total polysaccharide by the phenol-sulfuric said method and dinitrosalicylic acid colorimetric method.

### 4.3. Cell Viability Assay

When the growth density of LLC-PK1 in 75 flask reached 80–90%, trypsin was added to interact with the cells after washing with PBS. After the cells were dispersed, the number of cells was counted with a hemocytometer. RPMI-1640 containing 10% (*v*/*v*) FBS and 1% penicillin was put in 96-well of cell culture plates, and 10 μL of FBS-containing culture medium was added to each well. After culturing for 24 h and after the cells were adsorbed to the bottom, the culture medium was removed, and STZ was added to stimulate oxidative stress, and then, the culture was continued for 48 and 72 h. An amount of 20 uL MTT solution (dissolved in 5 mg/mL PBS) was added per well, and the wells were put into a carbon dioxide incubator with 5% CO2 at 37 °C and a constant temperature of 90% to react with cells for 4 h. Then, the solution in each well was poured out, followed by adding 100 uL DMSO to dissolve the blue-violet crystals in each well and protect them from light for about 10 min. The 96-well plates were shaken evenly to ensure that the blue-violet crystals are completely dissolved, and the absorbance was read at a wavelength of 570 nm with an enzyme immunoassay reader. Because only living cells have active mitochondrial dehydrogenase, the measured light absorbance was proportional to cell viability. The higher the reading, the greater the relative number of living cells. In the experimental framework of the above cell viability, the time points were set as the first day and the third day, and two concentrations of STZ (10 μm) were used to destroy renal tubular cells. The other drug concentrations were ACEI (1 mg/mL and 100 μg/mL), ARB (1 mg/mL and 100 μg/mL) and EtCE (1 mg/mL and 100 μg/mL), EtCE-EA (1 mg/mL and 100 μg/mL), EtCE-nB (1 mg/mL and 100 μg/mL), EtCE-W (1 mg/mL and 100 μg/mL), and HWCE (1 mg/mL and 100 μg/mL). The third day of cell culture from the 96-well plate was the zeroth day of the above experimental design. After removing the culture medium and drugs of various concentrations were added, the experiment started, and each culture time point was reached after collecting the data.

### 4.4. Animal Preparation

Female CRF mice, 6 weeks of age, were purchased from the BioLASCO Taiwan Co., Ltd. (Nangang, Taipei, Taiwan). All animals were maintained in laminar flow cabinets with free access to food and water under specific pathogen-free conditions in facilities approved by the Accreditation of Laboratory Animal Care and the Institutional Animal Care and Use Committee (IACUC) of the Animal Research Committee of the Southern Taiwan University of Science and Technology, Tainan, Taiwan (Approval No. STUT-IACUC-98-05). Five mice per cage were fed with mouse chow and water ad libitum. The mice were acclimatized to the 12/12 h light-dark cycle conditions in the cages and were kept in the housing facility for a 1-week acclimation period before the surgical injury. After the experimental animals were stably raised for two weeks, the kidneys (1/3 NT and 5/6 NT residual kidney) were removed in vivo. Each experimental mouse was anesthetized by intraperitoneal injection, and the dose of anesthetic was 0.01 c.c. Through back surgery, the unilateral kidney was divided into three equal parts, the upper and lower parts were sutured, and antibiotics were applied to the wound to avoid infection and death. The survival rate and postoperative recovery were recorded.

The experimental animals were stably raised for two weeks and underwent Sham-operated kidney excision surgery. Each mouse was anesthetized with an intraperitoneal injection with an anesthetic dose of 0.01 c.c. From the back operation, the unilateral kidney was taken out of the abdominal cavity and put back and sutured without harming the kidney, and antibiotics were applied to the wound to avoid infection and death; the mice were observed for 7 days, and the survival rate and postoperative recovery were recorded. After the experimental animals were stably reared for two weeks and fed a normal diet, they were given a high dose of STZ 100 mg/kg/7 days, and middle doses of STZ 75 mg/kg/7 days were injected intraperitoneally to induce renal lesions (chemically-induced chronic nephropathy). Their survival was recorded, and weight monitoring was done weekly. After the experimental animals were established with 1/3 NT residual kidney animal type, they were given a high dose of STZ 100 mg/kg/7 days and a middle dose of STZ 75 mg/kg/7 days 1/3 NT plus STZ-induced chronic renal failure model.

The study group consisted of normal control, Sham group, 1/3 NT, STZ 75 mg/kg/7 days/i.p., STZ 100 mg/kg/7 days/i.p., 1/3 NT + STZ 100 mg/kg/7 days/i.p., and 1/3 NT+ STZ 100 mg/kg/7 days i.p. According to each time point before and after surgery and before and after administration of high-dose STZ, urine protein content was detected by the metabolic cage method. At each time point, the mice were treated with STZ before and after treatment. One week after induced STZ, the laboratory animals were stimulated to urinate, and the urine was collected and sent to the medical laboratory for testing. Normal control, Sham group, 1/3 NT, STZ 75 mg/kg/7 days/i.p., STZ 100 mg/kg/7 days/i.p., 1/3 NT + STZ 100 mg/kg/7 days/i.p., 1/3 NT + STZ 100 mg/kg/7 days i.p. blood collection was performed at each time point before and after surgery and before and after administration of high-dose STZ for the relevant biochemical value detection. If the animal did not need anesthesia or restraint with a restraint device, blood collection from the eye socket was used to confirm. After there was blood flowing out, about 0.5 mL of whole blood was collected with a blood collection tube, and after centrifugation at 5000 rpm for 10 min, 0.2 mL of serum was taken for biochemical value experiments. Blood biochemical values measured were creatinine (Cre), blood urea nitrogen (BUN). During the experiments, we recorded the survival rate and weekly body weight measurement of each mouse.

### 4.5. Blood Biochemical Profile

All groups’ blood samples were collected from the tail vein. FBG was measured with glucose oxidase strips (Easytouch, Taipei, Taiwan). Sham group, 1/3 NT + STZ group, 1/3 NT + STZ + ACEI (20 mg/kg) group, 1/3 NT + STZ + EtCE-W/EA group (100 mg/kg, 300 mg/kg and 500 mg/kg), and 1/3 NT + STZ + HWCE group (500 mg/kg) were continuously administrated for 2 weeks. According to each time point before and after surgery and before and after administration of high-dose STZ, urine protein content was detected by the metabolic cage. At each time point, the mice were treated with STZ before and after treatment. One week after induced STZ, the laboratory animals were stimulated to urinate, and the urine was collected and sent to the medical laboratory for testing. Blood collection was performed at each time point before and after surgery and before and after administration of high-dose STZ for relevant biochemical value detection. If the animal did not need anesthesia or restraint with a restraint device, blood collection from the eye socket was used to confirm. After there was blood flowing out, about 0.5 mL of whole blood was collected by a blood collection tube, and after centrifugation at 5000 rpm for 10 min, 0.2 mL of serum is taken for biochemical value experiments. Blood biochemical values (Glucose, Albumin-Creatinine Ratio (%), blood urea nitrogen (BUN), Creatinine (Cre)) were measured during the experiments. Blood serum metabolic enzymes were quantified using an enzyme-linked immunosorbent assay (ELISA).

### 4.6. Hematoxylin and Eosin (HE)

After mice were sacrificed, the excised kidney samples were fixed in formalin. The samples were dehydrated through a gradient mixture of ethyl alcohol and water, then rinsed with xylene before being embedded in paraffin. The formalin fixed tissues were sliced in 5 μm sections using a Microtome RM2135 (Leica Microsystems Inc., Bannockburn, IL, USA) and prepared on silane-coated slides. The slides were immersed in Tris-buffered saline (TBS, pH 7.4) after being rehydrated in graded ethanol solutions, dried at 37 °C overnight, and then stored at room temperature. The 5 μm kidney sections were stained with hematoxylin (Shandon™ Gill™ III) and Shandon Eosin Y (Thermo Scientific™). Lastly, the slides underwent microscopic examination by means of a Motic BA 400 microscope with Motic Advance 3.0 software (Motic Co., Fujian, China).

### 4.7. Masson Trichrome Staining

Kidney samples were fixed in 10% formal-saline for 48 h and then dehydrated by successively passing through a gradient of mixtures of ethyl alcohol and water. The samples were then rinsed with xylene and embedded in paraffin. Kidney sections (5 μm thick) were prepared and stained with Dietrich scarlet-acid fuchsin solution for 15 min, then transferred directly to aniline blue solution and stained for 5–10 min. Finally, the sections were mounted using neutral deparaffinated xylene (DPX) medium for microscopic examination on a Motic BA 400 microscope using Motic Advance 3.0 software.

### 4.8. Immunohistochemical Stain

The kidney samples were fixed in formalin and dehydrated with a gradient mixture of ethyl alcohol and water. The samples were then rinsed with xylene and embedded in paraffin. The formalin-fixed tissues were sliced by Microtome RM2135 (Leica Microsystems Inc., Bannockburn, IL, USA) into 5 μm sections and placed on silane-coated slides. The slides were immersed in Tris-buffered saline (TBS, pH 7.4) after being rehydrated in graded ethanol solutions, dried at 37 °C overnight, and stored at room temperature. After that, the sections were soaked in 0.3% H2O2 to block the endogenous peroxidase activity. They were then placed in the 10 mM citrate buffer solution (pH = 6.0) and microwave boiled for 10 min for completing antigen retrieval. The sections were incubated with primary antibodies against TGF-β and α-SMA (1:250 dilution) in a humidified chamber at room temperature for 2 h. The LSAB2 detection and DAB substrate kits were used for staining processes according to the manufacturer DAKO’s instructions. Finally, the sections were counterstained with hematoxylin (Shandon™ Gill™ III) and the number of stained nuclei (dark blue color) per square millimeter was calculated using an eyepiece graticule. For the positive labeling index for TGF-β and α-SMA, each tissue slide was illustrated as an average percentage of dividing the numbers of a TGF-β and α-SMA-positive cell (visualized in brown) by the total numbers of nuclei (visualized in blue). With each staining run, both positive and negative controls were provided, and overexpression was considered positive if more than 10% of the cells were showing.

### 4.9. Statistical Analysis

All the results were presented as the mean ± standard deviation (SD). Differences between groups were evaluated with an analysis of variance and post hoc comparisons with the Bonferroni step-down (Holm) correction. Statistical analysis was performed using SigmaPlot software (version 10.0; SPSS Inc., Chicago, IL, USA). Post hoc testing of behavioral data utilized a two-tailed Welch’s *t*-test. Post hoc testing of biochemical data utilized a regression analysis. Each value represents the mean ± SD of 8 mice; *p* values less than 0.05 were considered statistically significant. The values * *p* < 0.05, ** *p* < 0.01, and *** *p* < 0.001 represent significant differences between the vehicle control and normal group (drinking water alone), and ^#^ *p* < 0.05, ^##^ *p* < 0.01, and ^###^ *p* < 0.001 represent significant differences from the 1/3 NT group and same dose of STZ.

## Data Availability

No new data were created or analyzed in this study. Data sharing is not applicable to this article.
